# 

**Published:** 1903-02-07

**Authors:** 


					^ CADBURY'S ^ 'ff L"' CADBURYS
COCOA jJJ 1^* | ~ COCOA
ABSOLUTELY PURE. ^ NO DRUGS OR ALKALIES USED.
No. 854. Vol. XXXIII. C?ww?]
Saturday, Feb. 7th, 1903.
[SubscriptionTerms,] pRICH TWOPENCE.

				

